# Characteristics of Artificial Intelligence Clinical Trials in the Field of Healthcare: A Cross-Sectional Study on ClinicalTrials.gov

**DOI:** 10.3390/ijerph192013691

**Published:** 2022-10-21

**Authors:** Anran Wang, Xiaolei Xiu, Shengyu Liu, Qing Qian, Sizhu Wu

**Affiliations:** Department of Medical Data Sharing, Institute of Medical Information & Library, Chinese Academy of Medical Sciences & Peking Union Medical College, Beijing 100020, China

**Keywords:** artificial intelligence, healthcare, clinical trials, registry analysis, ClinicalTrials.gov

## Abstract

Artificial intelligence (AI) has driven innovative transformation in healthcare service patterns, despite a lack of understanding of its performance in clinical practice. We conducted a cross-sectional analysis of AI-related trials in healthcare based on ClinicalTrials.gov, intending to investigate the trial characteristics and AI’s development status. Additionally, the Neo4j graph database and visualization technology were employed to construct an AI technology application graph, achieving a visual representation and analysis of research hotspots in healthcare AI. A total of 1725 eligible trials that were registered in ClinicalTrials.gov up to 31 March 2022 were included in this study. The number of trial registrations has dramatically grown each year since 2016. However, the AI-related trials had some design drawbacks and problems with poor-quality result reporting. The proportion of trials with prospective and randomized designs was insufficient, and most studies did not report results upon completion. Currently, most healthcare AI application studies are based on data-driven learning algorithms, covering various disease areas and healthcare scenarios. As few studies have publicly reported results on ClinicalTrials.gov, there is not enough evidence to support an assessment of AI’s actual performance. The widespread implementation of AI technology in healthcare still faces many challenges and requires more high-quality prospective clinical validation.

## 1. Introduction

Artificial intelligence (AI) development in the healthcare industry has a solid technical foundation due to the rapid growth of information technology and the accumulation of massive biomedical data. Researchers are actively exploring AI applications to improve the efficiency of medical diagnosis and service quality and to reduce complexity and risk [1,2]. The integration of AI technologies, represented by machine learning (ML), neural networks (NN), deep learning (DL), natural language processing (NLP), and computer vision (CV), with the healthcare field has continuously deepened, presenting diversified developmental patterns, such as the following:

- Medical image processing, e.g., automatic segmentation, classification, and feature recognition of pathological and cellular images based on ML and DL [3,4,5];
- Diagnosis and prediction, e.g., enhancing the accuracy of disease diagnosis and improving patient prognosis prediction through AI technology [6,7,8,9];
- Clinical decision support, e.g., integrating NLP into electronic health records (EHRs) to identify errors and omissions in treatment protocols and provide more effective therapy for patients [10,11] and performing patient risk stratification based on artificial neural networks (ANN) to improve emergency triage efficiency [12];
- Patient monitoring and management, e.g., combining AI technology with mobile medical devices and the Internet of Things to facilitate remote patient monitoring and medication adherence management [13,14,15,16];
- Medical robotics, e.g., providing surgical planning and surgical stage identification by DL and CV [17,18];
- Precision medicine, e.g., using AI to develop biomarkers and predict patient responses to treatment [19,20] and improving detection of tumors’ genetic variants through ML [21];
- Drug discovery and repurposing, e.g., optimizing drug structure design and predicting drug–target interactions based on AI to shorten new drug development time or identify new indications for known drugs [22,23].

In recent years, the number of medical AI-related publications has been increasing yearly [24]. It is worth noting that there is still a lack of comprehensive and quantitative research on the performance and impact of AI applications in the real-world medical environment. Undoubtedly, AI brings technological enhancements to healthcare processes, including diagnosis, treatment, and prognosis. Numerous studies have demonstrated that AI performs comparably to or better than humans in specific tasks [25,26,27]. However, there is a considerable dispute in the medical community about whether the prominent performance of AI under research conditions can be translated and deployed to the real-world medical environment. The few proven AI application examples based on clinical settings are not enough to support the widespread use and involvement of AI technology in the medical field [28]; hence, further clinical validation of AI applications is required.

Clinical trials are exploratory research oriented toward the frontiers of disciplines to find novel methods for disease diagnosis, treatment, and health improvement. Well-designed and executed clinical trials can significantly improve the effectiveness and efficiency of medical studies [29]. To enhance the transparency of clinical trials and lessen the bias of trial results, the research community has been devoted to promoting clinical trial registration [30]. ClinicalTrials.gov [31], jointly established by the United States Food and Drug Administration (FDA) and the National Library of Medicine (NLM), is the largest and most influential registry platform in the world and contains more than 420,000 trials registered from 221 countries and regions. Previous studies indicate that the published clinical trial literature may be biased toward positive or promising results [32,33], whereas studies based on clinical trial registration information are more useful for researchers investigating the development tendency in related technical fields and identifying shortcomings and neglected research directions.

Based on the ClinicalTrials.gov database, there have been many reports of tracking studies and feature analyses of clinical trials in different fields, such as ophthalmology [34], pediatrics [35], traditional Chinese medicine [36], infectious diseases [37,38], and digital health [39], revealing the heterogeneity of the test methodology. In the medical AI field, although Zippel et al. analyzed the ML (a subdomain of AI) applications in clinical research [40], Dong et al. and Liu et al. described the current status of registered trials for AI in cancer diagnosis [41], critical care, and emergencies [42], they have not yet conducted a comprehensive assessment of clinical trials related to AI and there is a lack of a thorough understanding of AI clinical trials.

The translation and application of new technologies into clinical practice is a long process. Based on the registered trials of the ClinicalTrials.gov database, this study attempts to describe the basic characteristics and application development of AI-related trials in the field of healthcare, and provides a reference for improving the design of AI clinical trials and promoting clinical transformation.

## 2. Materials and Methods

### 2.1. Data Search

We used the advanced search function of the ClinicalTrials.gov website [43] to retrieve AI-related trials in healthcare that were first posted before 31 March 2022. The searched words included Medical Subject Headings (MeSH) concepts about “Artificial Intelligence” [44], such as artificial intelligence, AI, computational intelligence, machine intelligence, computer reasoning, computer vision system, knowledge acquisition (computer), knowledge representation (computer), computer heuristics, expert systems, fuzzy logic, knowledge bases, machine learning, deep learning, support vector machine, natural language processing, neural networks of computer, robotics, sentiment analysis; and commonly used AI-related terms, including learning algorithm, supervised learning, unsupervised learning, reinforcement learning, pattern recognition, image recognition, machine perception, cognitive computing, autonomic computing, clustering, cluster analysis, decision tree, random forest, and genetic algorithm. A total of 8801 retrieved results were downloaded in XML file format on 21 April 2022.

### 2.2. Data Screening and Extraction

**Figure 1:**
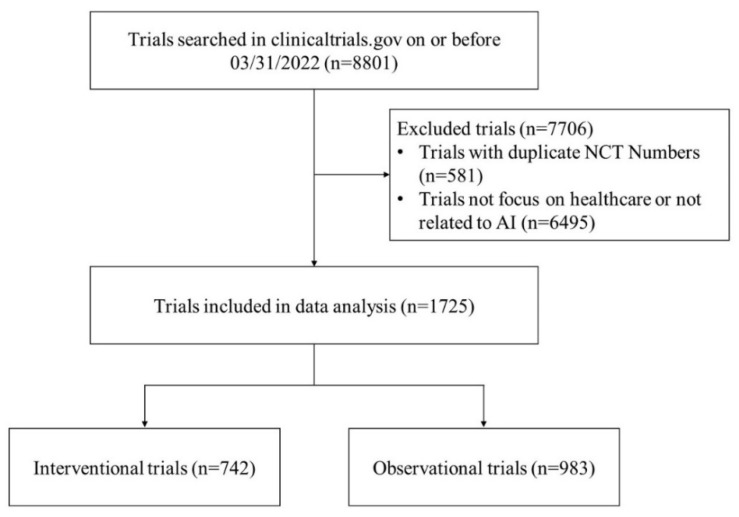
Flowchart of trial selection.

The selection process of trials is outlined in a flowchart (Figure 1). First, we removed 581 duplicate records based on NCT numbers. After that, we scanned and reviewed the text information of registered trials, including the titles, study descriptions, study designs, conditions, interventions, eligibility criteria, MeSH terms, and keywords. Trials unrelated to any AI technology or unapplied to healthcare settings were removed. Two researchers performed data screening independently, and a third reviewed any disagreements. We finally identified 1725 studies that met our inclusion criteria. The following information was extracted: registered number, study type, published date, recruitment status, study results, conditions, enrollment, participant gender, participant age, recruitment location, number of centers, lead sponsor, funding source, allocation, intervention model, masking, phases of the trial, observational model, and time perspective. Further manual classification of the “lead sponsor” and “recruitment location” fields was also performed.

### 2.3. Data Processing

In addition, this study focused on three categories of healthcare AI technology classification, disease classification of AI-related trials, and healthcare application scenario classification of AI-related trials to explore the research hotspots in the current field.

Healthcare AI technology classification: According to the definition of AI issued by the High-Level Expert Group on Artificial Intelligence (in the European Commission) [45], related AI technologies can be divided into three domains, which are (1) reasoning and decision-making, (2) learning and perception, and (3) integration (robotics).

- “Reasoning and decision-making” embodies the ability to transform data into knowledge and organize decisions, typically using symbolic rules for knowledge representation, reasoning, planning, and optimization;
- “Learning and perception” usually uses structured or unstructured data for problem-solving, including learning and perception of changes, and covers AI subdomains, such as ML, NN, DL, NLP, and CV;
- Robotics as “Embodied AI” is an integrated application of multiple AI system capabilities (reasoning, learning, and perception).

Referring to the above taxonomy [45] and the MeSH hierarchical structure of the “Artificial Intelligence” term [44], we preliminary classified the key AI-related concepts (Table 1) in a top-down manner. Then, the AI-related algorithms or models involved in the clinical trials were extracted from the textual information, such as the “study title”, “study description”, “outcome measures”, and “study-relevant MeSH terms”, through a combination of NLP and manual review to further improve and enrich the AI technology classification.

Disease classification of AI-related trials: the disease areas of AI-related trials were classified by searching for the terms provided in the “conditions” field with the online “ICD-11 for Mortality and Morbidity Statistics” [46], exploring the primary target population of current clinical trials.

Healthcare application scenario classification of AI-related trials: the application fields were classified by manually reviewing the textual information to investigate the topics of AI technology in healthcare focused upon.

### 2.4. Statistical Analysis

This study aimed to investigate the characteristics of registered trials for AI in healthcare, and a descriptive analysis was utilized to analyze the variables. The categorical variables were reported as frequencies and percentages, and the continuous variables were characterized as median and interquartile ranges (IQR). An additional stratification by key study features (study type and reporting results status) was conducted, and the Chi-square test or Fisher test (if the number of studies was <5) was used to compare proportions. Univariate and multivariable logistic regression models were used to determine independent risk factors associated with reporting results. Variables with *p* < 0.1 in the univariate analysis were subsequently included in a multivariable analysis, and the variance inflation factor was estimated to ensure the absence of multiple collinearities. Statistical analysis was performed using SPSS version 23.0 (IBM Corporation) and *p* < 0.05 was considered statistically significant.

In addition, through the above data classifications and processing, we obtained structured data about AI technologies, disease areas, and healthcare application scenarios related to the included clinical trials. To achieve a visual representation and analysis of clinical research hotspots, we further constructed an AI technology application graph based on the Neo4j graph database and visualization technology [47].

## 3. Results

### 3.1. Characteristics of the Included Trials

A total of 1725 trials were included—742 (43.01%) were interventional trials and 983 (56.99%) were observational trials (Table 2). Overall, 451 (26.14%) trials were already completed, 717 (41.57%) were in the process of recruiting, 323 (18.72%) were not recruiting, 6 (0.35%) were suspended, 26 (1.51%) were terminated, 29 (1.68%) were withdrawn, and 173 (10.03%) were unknown. Among the interventional studies, 391 (52.70%) trials were randomized, 92 (12.40%) were non-randomized, 257 (34.64%) were not applicable, and 2 (0.27%) were unknown. For assignment type, 398 (53.64%) trials had parallel assignment, 269 (36.25%) had single group assignment, 45 (6.06%) had crossover assignment, 19 (2.56%) had sequential assignment, 10 (1.35%) had factorial assignment, and 1 did not provide information. For masking, 477 (64.29%) were open-labeled, 141 (19.00%) were single-blind, 69 (9.30%) were double-blind, 38 (5.12%) were triple-blind, 15 (2.02%) were quadruple-blind, and 2 (0.27%) were unknown. Moreover, most trials did not mention the phases of the trials (90.44%). Among the observational trials, 594 (60.43%) were cohort studies, 124 (12.61%) were case-only studies, 116 (11.80%) were case–control studies, 14 (1.42%) were case–crossover studies, 129 (13.12%) were other types of studies, and 6 (0.61%) did not report information. A total of 589 (59.92%) were prospective studies, 236 (24.01%) were retrospective studies, 97 (9.87%) were cross-sectional studies, 58 (5.90%) were other designed studies, and 3 (0.31%) trials could not be classified due to the value being missing.

**Figure 2:**
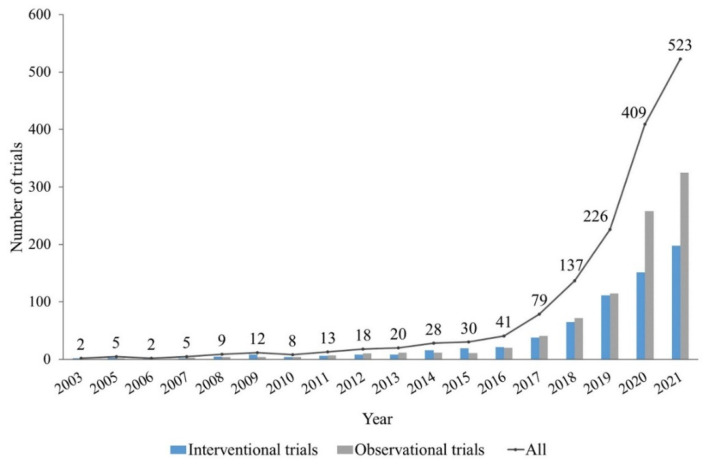
Distribution of AI-related trials according to the posted year on ClinicalTrials.gov (*n* = 1725).

The number of AI-related trial registrations in healthcare has grown dramatically each year since 2016 (Figure 2). In total, 1573 studies involving AI were conducted between 2016 and 2022, representing 91.19% of all trials. The stratified characteristics of the included trials are shown in Table 3. Only 30 (1.74%) trials reported results on ClinicalTrials.gov, with significant differences between the interventional and observational trials (3.77% vs. 0.20%, *p* < 0.001). Most trials were single-center (69.39%) and were conducted in Europe, Asia, and North America (94.20% of the interventional trials and 95.63% of the observational trials). For recruited samples, 80.06% of the trials consisted of adults and older adults and 92.41% did not restrict participants to gender. The median enrollment was 250 (IQR: 71.75–1000). More interventional trails than observational trials had fewer than 100 participants (47.44% vs. 23.91%), while observational trials had a higher proportion of groups consisting of 100–500 participants (33.47% vs. 30.46%), 500–1000 participants (14.34% vs. 7.41%), and more than 1000 participants (28.18% vs. 14.69%) (all *p* < 0.001). Most trials were sponsored by hospitals or universities (64.23%), while 15.94% were sponsored by industries and 19.83% were sponsored by other institutions or individuals. For funding sources, more interventional trials than observational trials were (co-) funded by the United States Federal Agencies/National Institutes of Health (11.19% vs. 4.58%) or industries (21.56% vs. 18.62%) (both *p* < 0.001).

We explored factors influencing study results reporting to provide an initial insight into the shortcomings of clinical trial results reporting and then further facilitate problem-solving. Given that a handful of observational trials reported results, univariate and multivariable logistic regression analyses were performed using the registered interventional trials. The distribution of key characteristics is shown in Table S1 (Supplementary Information). In multivariate logistic regression analysis, interventional trials with multicenter designs were more likely to report their results publicly on ClinicalTrials.gov (adjusted OR = 3.99; 95% CI: 1.54–10.29; *p* < 0.05) (Table 4).

### 3.2. Overview of AI Technology Applications in AI-Related Trials

As shown in Table 5, the categorization statistics revealed that ML was the most applied technology—664 (57.62%) studies described the detailed ML algorithms or models used in clinical trials, with NN and DL being the most prominent. In addition, 398 (23.13%) studies did not state the specific ML algorithms. Seventy-eight (4.52%) and fifty-nine (3.42%) studies used visual perception and language understanding algorithms, mainly in image recognition, CV, and NLP. Reasoning and decision-making technology was mentioned in 46 (2.67%) studies, and 111 (6.43%) focused on AI–robotics research.

### 3.3. Overview of Disease Areas in AI-Related Trials

AI-related trials in healthcare covered 24 disease areas (Table 6), with 152 (8.81%) trials not listing ICD-11 coded conditions (e.g., AI and healthy subjects). The most common areas were neoplasms (392, 24.92%), circulatory system diseases (204, 12.97%), nervous system diseases (181, 11.51%), digestive system diseases (139, 8.84%), and mental, behavioral, or neurodevelopmental disorders (136, 8.65%). In addition, 66 (4.20%) trials involved COVID-19 and post-COVID-19 condition studies.

### 3.4. Overview of Healthcare Application Scenarios in AI-Related Trials

The registered trials also targeted various healthcare application scenarios (Table 7). Among the 1725 studies, 662 (38.38%) trials focused on disease diagnosis and screening, followed by medical imaging (365, 21.16%), clinical outcome prediction (296, 17.16%), patient monitoring and management (177, 10.26%), clinical decision support (155, 8.99%), adjuvant treatment (108, 6.26%), surgery (95, 5.51%), and rehabilitation (78, 4.52%).

### 3.5. Construction of AI Technology Application Graph

We constructed an AI technology application graph based on the Neo4j graph database and visualization technology to investigate the application status of AI technology in various healthcare scenarios and discover the disease areas focused upon. Neo4j supports storing data as an “entity–relationship–entity” triple. We defined four types of entities, including the AI subdomains classification (AI_subdomain), detailed AI technologies in different subdomains (AI_technology), conditions classification (condition), and healthcare application setting classification (healthcare_setting), and three types of entity relationships. See Tables S2 and S3 (Supplementary Information) for a detailed description of the entities and entity relationships.

**Figure 3:**
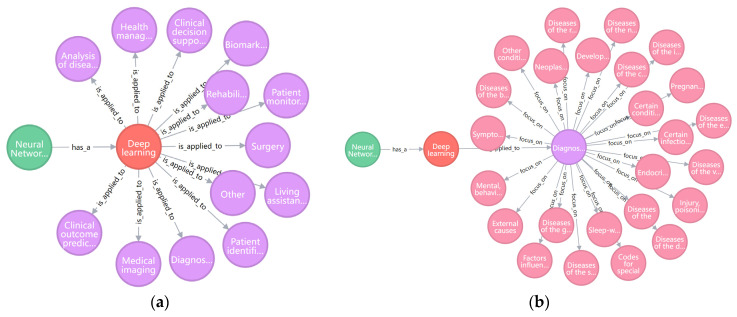
Part of the AI technology application graph. (**a**) The left figure shows the application of DL technology in different healthcare scenarios. (**b**) The right figure shows the situation of DL technology application in the diagnosis and screening field of various condition areas.

Part of the AI technology application graph is shown in Figure 3, demonstrating the application of DL technology in different healthcare scenarios and condition areas. Different colors distinguish entity categories, with green nodes indicating the AI_subdomain entity, orange nodes indicating the AI_technology entity, purple nodes indicating the healthcare_setting entity, and pink nodes indicating the condition entity. See Table S4 for statistics (Supplementary Information).

## 4. Discussion

To our knowledge, this study is the first comprehensive and quantitative evaluation of the characteristics of AI-related clinical trials in healthcare based on the ClinicalTrials.gov database. The results show the registered trials’ features and the healthcare AI technology’s development and application, help to specify the situation of clinical trial design in this field, and provide a reference for promoting the clinical application of AI technology.

### 4.1. Characteristics of AI-Related Trials

Up to March 2022, 1725 relevant clinical trials were published on ClinicalTrials.gov. The number of trial registrations began to increase exponentially in 2016, which might be attributed to the development of industry 4.0 and the beginning of countries paying attention to AI technology. Since 2016, the United States, France, Canada, China, and the United Kingdom have successively issued national AI strategies and have made key arrangements in medical care and healthfulness [48,49,50,51,52]. The results of the present study show that the recruitment locations for AI-related trials were mainly distributed in Europe, Asia, and North America. Among them, the United States initiated the most clinical trials (420, 24.35%), followed by China (358, 20.75%), France (125, 7.25%), the United Kingdom (109, 6.32%), and Canada (76, 4.41%) (Table 3). From the perspective of funding, the majority of trials were (co-)funded by hospitals, universities, or academic institutions (88.70%), but the proportion of trials (co-)funded by industries or companies was lower than the overall status of registered trials in ClinicalTrials.gov [53].

As a whole, AI-related clinical studies have some drawbacks regarding trial design. The existing studies were dominated by small single-center trials, with 77.57% of trials enrolling less than 1000 participants and 34.03% enrolling 100 or fewer participants. Most trials were observational designs (56.99%) and 40.08% of observational trials were non-prospective studies. A total of 47.30% interventional trials were non-randomized and only 35.40% used blinded designs, and their results were lower than those of all trials in the ClinicalTrials.gov database [53]. More than 90% of AI-related trials failed to be classified into conventional clinical trial staging, indicating that conventional clinical trial staging criteria and evaluation criteria might not entirely apply to the AI field.

AI-related clinical trials also suffer from poor-quality results reporting. The public reporting of study methods and outcomes can help to reduce disclosure bias and improve study effectiveness [54,55]. However, it was found that only 1.74% of trials reported results on ClinicalTrials.gov; there were significantly fewer observational trials than interventional trials, while most studies did not report results upon completion (427/451, 94.68%). The proportion of results reporting for AI-related trials was lower than the overall statistics of registered studies in ClinicalTrials.gov [56,57], suggesting that improvements in trial transparency are needed. Furthermore, the analysis of factors associated with reporting results revealed that interventional trials with multicenter design were more likely to publish study results on ClinicalTrials.gov. Several previous studies have also validated our findings. In the research of characteristics among randomized controlled trials (RCTs) in gastric cancer, Lu et al. noted that RCTs conducted in non-Asian regions (OR = 2.76; 95% CI: 1.03–7.35) or with multicenter designs (OR = 3.84; 95% CI: 1.58–9.38) or external funds (OR = 2.373; 95% CI: 1.05–5.38) were more likely to publish their outcomes [58]. A retrospective study of the literature by Canestaro et al. also found that trials with multicenter designs (OR = 1.32; 95% CI: 1.16–1.45) were associated with study results publication [59].

### 4.2. Status of AI Application

It was found that the current research perspective focuses more on disease diagnosis and screening based on ML, DL, convolutional neural networks (CNNs), and CV technologies. Among them, the higher proportion of disease areas involves neoplasms (especially adenoma, colorectal tumors, breast cancer, and skin cancer), digestive system diseases (especially polyps), and visual system diseases (especially diabetic retinopathy). In recent years, ML, DL, and CV have attracted growing attention due to the visual pattern-recognition nature of diagnostic tasks in some specialized areas, such as radiology, pathology, endoscopic imaging, ophthalmology, and dermatology [60,61]. These technologies were commonly trained using manually annotated structured or unstructured datasets, achieving lesion recognition or disease classification. Theoretically, the diagnostic accuracy of AI systems can be comparable to or better than that of professional physicians if sufficient data are available. This view has been supported by the results of many previous studies under retrospective settings [25,26,27]. Nonetheless, the performance and impact of AI in real-world clinical settings still require further validation. In our study, an interventional trial (NCT04693078) was performed to explore whether the Detection of Elusive Polyps system (DEEP) based on DL could alert the existence and position of polyps in real-time. The results demonstrated that the detection system could effectively improve the polyp detection rate and reduce false alarms [62]. There were also some registered trials in ClinicalTrials.gov dedicated to investigating whether the diagnostic performance of AI could be superior to that of clinical experts (e.g., NCT04671368, NCT04040374, and NCT04562168). However, the study results have not been reported.

AI technology also has great potential for clinical outcome prediction, patient monitoring and management, and adjunctive treatment. The combination of ML, especially supervised learning and DL, with various types of medical data (e.g., EHR, medical images, and physiological signals) has yielded remarkable results in clinical prediction (e.g., disease recurrence risk, therapeutic response, therapeutic effect, adverse outcomes, readmission rates, hospitalization time, and mortality). Meanwhile, AI technology has supported the continuous innovation of wearable devices and mobile health tools. (Un-)supervised learning, reinforcement learning (RL), and NLP have had many achievements in patient remote monitoring and management, patient intervention and treatment, and health promotion. Several studies with reported results were worthy of our attention. A prospective interventional trial (NCT03643692) validated the ability of mobile apps embedded with DL algorithms to mitigate the risk of severe complications and enhance self-management for patients with type I diabetes. The results indicated that programs could achieve real-time glucose level prediction and early warning (the Matthews correlation coefficients of hypoglycemia and hyperglycemia prediction are 0.56  ±  0.07 and 0.70  ±  0.05) [63]. Another two comparative effectiveness studies aimed to confirm the feasibility and effectiveness of the mobile programs in the intervention treatment of depression and anxiety disorders (NCT02176226) and chronic pain (NCT02464449). The studies found that the introduction of ML and RL algorithms could facilitate the personalized formulation and adjustment of patient interventions, significantly improve patient compliance, and reduce the treatment time and labor costs compared with traditional treatment [64,65,66].

Medical robotics has reached a mature level in recent years. The present study data indicated that clinical trials on different types of medical robots, such as chatting robots, rehabilitation training robots, assistive wearable robots, and surgical robots, have been conducted for various research purposes involving design development, application testing, performance verification, and evaluation, for instance, feasibility and safety. Chatbots are a tangible manifestation of language-understanding technology that perform an essential role in patient management, disease detection, and health intervention through providing intelligent Q&A and interactions. A randomized controlled trial (NCT03556813) assessed the noninferiority of chatbots in delivering information to breast cancer patients, showing a higher success rate compared with that of physicians (69% vs. 64%, *p* < 0.001) [67]. Research on rehabilitation training robots focused on providing repetitive movement therapy, such as limb intelligent feedback training and adaptive gait training, for patients with neuromuscular injuries, most commonly stroke and spinal cord injury (e.g., NCT03057652 and NCT05238389). Assistive wearable robots, including powered prostheses and exoskeletons, were designed to improve the mobility of patients with limb amputations or neuromuscular impairment (e.g., NCT03264235 and NCT02274532). The development of AI has promoted the improvement of the functions of rehabilitation and assistive robots in biological signal recognition processing, pattern recognition control, and perception of motion intention. In addition, more than half of the trials of medical robotics focused on the surgery area. The combination of robotics, DL, and CV provides many assistive functions, such as preoperative planning, intraoperative imaging, and image guidance, for laparoscopic surgery, minimally invasive catheter surgery, joint-replacement surgery, and joint-correction surgery. A cohort study of autonomous robotics for transcatheter cardiac procedures was published on ClinicalTrials.gov in 2021 (NCT04787757) using 3D echocardiography, CT images, and AI algorithms to provide autonomous catheter navigation. Autonomy is a higher manifestation of the value of medical robotics [68]. Still, there are relatively few clinical trials on autonomous robotics, which may be due to the intricacy of artificial intelligence surgery [69], medical ethics, and legal supervision [70].

### 4.3. Challenges

In the traditional medical pattern, insufficient medical resources, long physician training cycles, and high clinical misdiagnosis rates are the pain points in healthcare [71,72]. AI’s potent data-processing capabilities and automated workflows can simplify complex and time-consuming medical tasks, rapidly integrate various medical information, and even enable real-time clinical decision-making. AI technology can perform a vital role in bridging resource shortages, reducing personnel costs, and improving the efficiency and accuracy of treatment. However, there are still many challenges to overcome before AI technology is widely implemented in real-world healthcare settings.

Standardization and privacy security of medical data are fundamental challenges for AI implementation. The majority of AI application studies focus on data-driven learning algorithms; therefore, the quality and quantity of medical data are essential for improving the effectiveness and performance of AI. However, there is significant structural variation in data across medical systems due to the absence of standardized guidance frameworks for EHR collection. The medical big data interoperation and sharing from different resources also present an ethical challenge [73,74]. Moreover, large-scale medical data may also face the risk of leakage during use, adversely affecting personal privacy [75]. It becomes clear that achieving secure and controlled aggregation, access, and utilization of medical big data is a pressing issue in this area.

The explainability of AI technology is a critical issue that has plagued the medical community, and some studies have argued that ignoring AI’s explainability could threaten the medical process [76,77]. Despite the remarkable performance of AI algorithms, such as DL, their decision-making process is similar to a “black box” that does not provide a plausible explanation for the decision outcome, especially when the AI conclusion differs from the physician’s judgment. In recent years, “Explainable AI (XAI)” has become a popular research area. The Defense Advanced Research Projects Agency (DARPA) launched the XAI program in 2017, intending to create new or modified ML technologies and produce explainable models that enable users to understand, trust, and effectively manage AI systems [78]. In 2018, the General Data Protection Regulation (GDPR) of the European Union also stated that data subjects have a right to request explanations about automated decisions made by algorithms [79]. Although several clinical trials have also worked on developing and validating model-explainable functions to increase confidence in clinical solutions (e.g., NCT04918602, NCT05009251, NCT05020626, and NCT05384002), the number of such cases remains small. At the moment, more effort is needed for developers to balance AI performance and transparency to build trust between multiple users and AI systems, including physicians, patients, and review departments.

There is the issue of clinical validation. Although trained models based on manually annotated datasets exhibit capabilities comparable to those of professional physicians in specific tasks, medical AIs’ performance has been exaggerated in the media, and they may perform worse in real-world situations [28,80]. AI systems require prospective clinical validation to demonstrate their impact on health outcomes. At the current stage, researchers have conducted numerous clinical studies around AI application development, testing, and verification, evaluating various aspects of feasibility, efficacy, safety, and accuracy. However, according to the registered trials, it was found that the proportion of AI clinical trials with prospective and randomized designs was insufficient, and many had design defects. Meanwhile, only a handful of clinical trials have reported study results after completion, making it impossible to determine the actual effectiveness of the technology in question. In 2020, two significant guidelines, SPIRIT-AI [81] and CONSORT-AI [82], were issued to standardize the study design and reporting of AI clinical trials and to enhance the integrity and quality of trial protocols. However, these two guidelines are the minimum for AI reporting, and there are other AI-related considerations that may be worth considering. AI is a rapidly evolving field, and as technology and novel applications develop, a range of guidelines and regulations must be updated and improved. More high-quality prospective clinical trials are also needed to explore the long-term impact of AI technology in real-world environments and provide available evidence for clinical translation.

### 4.4. Limitations

This study was conducted in the context of current healthcare AI applications; therefore, the following limitations should be acknowledged.

First, the dataset of our study corresponds to a subset of AI-related clinical trials, considering that Clinicaltrials.gov is not the only clinical trial registry. However, as ClinicalTrials.gov is the most prominent and influential registry platform for clinical trials in the world, findings based on it can represent the field’s current status.

Second, as ClinicalTrials.gov recommends, but does not mandate, the use of study-specific MeSH terms for trial registration, the searched words for this study referenced the MeSH concepts of AI and some commonly used terms, which may lead to the underestimation of the amount of research in this field. Simultaneously, when classifying AI algorithms or models, some clinical trials did not describe the specific type, only “AI system”, “ML algorithm”, etc., limiting the finer-grained AI technology classification statistics.

Third, relatively few interventional trials have been registered and completed in the healthcare AI field at this stage. When conducting association analyses between the characteristics of clinical trials and results reporting, some characteristics (e.g., masking, trial phases) were not included due to data distribution issues. However, the purpose of our study was to provide insight into the shortcomings of trial design and results reporting in this area and then further facilitate problem-solving.

Finally, most of the analyzed trials were without results reporting; therefore, our study lacks the quantitative analysis of clinical trial results. As the reporting of clinical trial results will continue to improve over time with the development of related intervention policies, we believe that more attention should be paid to tracking and evaluating AI studies continuously.

## 5. Conclusions

This study provided a comprehensive quantitative assessment of AI-related trials in healthcare based on the ClinicalTrials.gov database. The results demonstrated the characteristics of registered trials and the development and application situations of AI technology in healthcare. Significant findings include the following:

First, the number of AI-related trials in the healthcare field registered on ClinicalTrials.gov has grown exponentially since 2016. Most trials were conducted in Europe, Asia, and North America, with the United States and China initiating the most studies. Hospitals and universities were the primary sponsors, but the proportion of trials sponsored by industries was relatively lower than that in other areas. Second, from the perspective of trial design, the AI-related clinical studies were dominated by small single-center trials, the proportion of trials with prospective and randomized designs was insufficient, and most studies did not report results upon completion. The overall standardization and transparency of clinical trials need to be improved. In addition, most healthcare AI application studies primarily focused on data-driven learning algorithms, covering various disease areas and healthcare scenarios. As only a few studies have publicly reported results on ClinicalTrials.gov, there is not yet enough to support an assessment of the actual performance of AI in healthcare. Finally, there are still many challenges to the widespread implementation of AI technology, and the field requires specific regulation and evaluation policies, and high-quality prospective clinical validation.

## Figures and Tables

**Table 1:** The taxonomy of AI.

AI Domain	AI Subdomain
Reasoning and decision-making	Computer reasoning
	Computer heuristics
	Fuzzy logic
	Expert systems
	Knowledge bases
Learning and perception	**Machine learning**
	Supervised machine learning
	Unsupervised machine learning
	Reinforcement learning
	Neural networks, including deep learning
	**Perception**
	Natural language processing, including sentiment analysis
	Computer vision
Integration	Robotics

**Table 2:** Study design elements of AI-related trials registered in ClinicalTrials.gov.

Characteristics	Number of Trials	Percent (%)
**All (*N* = 1725)**		
**Study type**		
Interventional	742	43.01
Observational	983	56.99
**Status**		
Completed	451	26.14
Recruiting	717	41.57
Not recruiting	323	18.72
Suspended	6	0.35
Terminated	26	1.51
Withdrawn	29	1.68
Unknown status	173	10.03
**Interventional (*n* = 742)**		
**Allocation**		
Randomized	391	52.70
Non-randomized	92	12.40
NA	257	34.64
Unknown	2	0.27
**Intervention model**		
Single group assignment	269	36.25
Parallel assignment	398	53.64
Crossover assignment	45	6.06
Sequential assignment	19	2.56
Factorial assignment	10	1.35
Unknown	1	0.13
**Masking**		
Open label	477	64.29
Single	141	19.00
Double	69	9.30
Triple	38	5.12
Quadruple	15	2.02
Unknown	2	0.27
**Phase**		
Phase 1	14	1.89
Phase 1/Phase 2	8	1.08
Phase 2	20	2.70
Phase 2/Phase 3	3	0.40
Phase 3	9	1.21
Phase 4	16	2.16
NA	672	90.57
**Observational (*n* = 983)**		
**Observational model**		
Case–control	116	11.80
Case–crossover	14	1.42
Case-only	124	12.61
Cohort	594	60.43
Other	129	13.12
Unknown	6	0.61
**Time perspective**		
Cross-sectional	97	9.87
Prospective	589	59.92
Retrospective	236	24.01
Other	58	5.90
Unknown	3	0.31

Abbreviations: NA, not applicable.

**Table 3:** Characteristics of AI-related trials stratified by study design.

Characteristics	Number (%) of Trials **			*p*-Value
	All (*N* = 1725)	Interventional (*n* = 742)	Observational (*n* = 983)	
**Posted Year**				
Before 2015	152 (8.81)	83 (11.19)	69 (7.02)	<0.001
2016	41 (2.38)	21 (2.83)	20 (2.03)	
2017	79 (4.58)	38 (5.12)	41 (4.17)	
2018	137 (7.94)	65 (8.76)	72 (7.32)	
2019	226 (13.10)	111 (14.96)	115 (11.70)	
2020	409 (23.71)	151 (20.35)	258 (26.25)	
2021	523 (30.32)	198 (26.68)	325 (33.06)	
2022	158 (9.16)	75 (10.11)	83 (8.44)	
**Study results**				
Has available results	30 (1.74)	28 (3.77)	2 (0.20)	<0.001
No available results	1695 (98.26)	714 (96.23)	981 (99.80)	
**Enrollment**				
≤100	587 (34.03)	352 (47.44)	235 (23.91)	<0.001
100–500	555 (32.17)	226 (30.46)	329 (33.47)	
500–1000	196 (11.36)	55 (7.41)	141 (14.34)	
>1000	386 (22.38)	109 (14.69)	277 (28.18)	
Unknown	1 (0.06)	0 (0)	1 (0.10)	
**Age group**				
Children only (<18 y)	59 (3.42)	33 (4.45)	26 (2.64)	<0.001
Adults only (18–65 y)	69 (4.00)	43 (5.80)	26 (2.64)	
Older adults only (>65 y)	29 (1.68)	16 (2.16)	13 (1.32)	
Children and adults	52 (3.01)	18 (2.43)	34 (3.46)	
Adults and older adults	1283 (74.38)	570 (76.82)	713 (72.53)	
All	233 (13.51)	62 (8.36)	171 (17.40)	
**Gender**				
Female only	108 (6.26)	45 (6.06)	63 (6.41)	0.862
Male only	23 (1.33)	11 (1.48)	12 (1.22)	
Both	1594 (92.41)	686 (92.45)	908 (92.37)	
**Center**				
Single-center	1197 (69.39)	507 (68.33)	690 (70.19)	0.699
Multi-center	330 (19.13)	146 (19.68)	184 (18.72)	
Unknown	198 (11.48)	89 (11.99)	109 (11.09)	
Region *				
Europe	622 (36.06)	231 (31.13)	391 (39.78)	
North America	498 (28.87)	283 (38.14)	215 (21.87)	
Asia	519 (30.09)	185 (24.93)	334 (33.98)	
Africa	29 (1.68)	12 (1.62)	17 (1.73)	<0.001
South America	23 (1.33)	8 (1.08)	15 (1.53)	
Oceania	18 (1.04)	12 (1.62)	6 (0.61)	
Unknown	192 (11.13)	87 (11.73)	105 (10.68)	
Country *				
United States	420 (24.35)	243 (32.75)	177 (18.01)	<0.001
China	358 (20.75)	109 (14.69)	249 (25.33)	
France	125 (7.25)	49 (6.60)	76 (7.73)	
United Kingdom	109 (6.32)	35 (4.72)	74 (7.53)	
Canada	76 (4.41)	38 (5.12)	38 (3.87)	
Italy	67 (3.88)	25 (3.37)	42 (4.27)	
Spain	50 (2.90)	27 (3.64)	23 (2.34)	
Germany	50 (2.90)	18 (2.43)	32 (3.26)	
Republic of Korea	41 (2.38)	17 (2.29)	24 (2.44)	
Switzerland	35 (2.03)	10 (1.35)	25 (2.54)	
All others	378 (21.91)	160 (21.56)	218 (22.18)	
Unknown	192 (11.13)	87 (11.73)	105 (10.68)	
**Lead sponsor**				
Hospital	614 (35.59)	236 (31.81)	378 (38.45)	
University	494 (28.64)	227 (30.59)	267 (27.16)	<0.05
Industry	275 (15.94)	125 (16.85)	150 (15.26)	
Other	342 (19.83)	154 (20.75)	188 (19.13)	
Funded by *				
NIH	95 (5.51)	63 (8.49)	32 (3.26)	<0.001
US Fed	33 (1.91)	20 (2.70)	13 (1.32)	
Industry	343 (19.88)	160 (21.56)	183 (18.62)	
Other	1530 (88.7)	654 (88.14)	876 (89.11)	

* More than one choice possible. ** Sum partly ≠ 100 due to multiple choices. Abbreviations: NIH, National Institutes of Health; US Fed, other United States Federal Agencies.

**Table 4:** Adjusted logistic regression analysis of the association of characteristics with results reporting.

Characteristics	Univariate Analysis		Multivariate Analysis	
	OR (95% CI)	*p*-Value	OR (95% CI)	*p*-Value
**Posted year**				
Before 2012 (reference)	1.00			
After 2013	0.26 (0.10–0.67)	<0.05	0.41 (0.16–1.42)	0.158
**Allocation**				
Randomized (reference)	1.00			
Non-randomized	2.12 (0.51–8.83)	0.300	2.63 (0.57–12.26)	0.218
NA	3.26 (1.29–8.21)	<0.05	2.69 (0.99–7.30)	0.052
**Masking**				
None/open label (reference)	1.00			
Single	0 (0)	0.997		
Double or more	0 (0)	0.998		
**Enrollment**				
≤100 (reference)				
>100	0.90 (0.39–2.05)	0.794		
**Center**				
Single-center (reference)	1.00			
Multi-center	5.54 (2.35–13.05)	<0.001	3.99 (1.54–10.29)	<0.05
**Lead sponsor**				
Other (reference)	1.00			
Industry	0.91 (0.31–2.74)	0.873		
University/Hospital	0.52 (0.19–1.41)	0.198		
**Region of lead sponsor**				
Asia (reference)	1.00			
North America	9.49 (1.24–72.45)	<0.05	5.49 (0.68–44.21)	0.110
Europe	2.31 (0.23–22.94)	0.474	1.79 (0.18–18.28)	0.623
Other	9.40 (0.51–174.48)	0.133	7.94 (0.35–178.58)	0.192
**Funded by**				
Industry/other (reference)	1.00			
NIH/US Fed	5.08 (1.94–13.30)	<0.05	1.77 (0.50–6.29)	0.376

Abbreviations: NA, not applicable; NIH, National Institutes of Health; US Fed, other United States Federal Agencies; OR, odds ratio; CI, confidence interval.

**Table 5:** AI technology applications in healthcare.

Domain	Subdomain (*n*) *		Terms (*n*) *	
Reasoning and decision-making	Computer reasoning (10)		Case-based reasoning (4)	
			Reasoning (6)	
	Computer heuristics (3)		Heuristics (3)	
	Fuzzy logic (3)		Fuzzy logic (3)	
	Expert systems (22)		Expert systems (22)	
	Optimization (8)		Genetic algorithm (8)	
Learning and perception	Machine learning	Supervised machine learning (191)	Classification	Decision tree (33)
				Support vector machine (42)
				Random forests (36)
				Boosting (12)
				Classifier not specified (34)
			Regression	Linear regression (15)
				Logistic regression (44)
				Regression models not specified (16)
			Supervised learning not specified (17)	
		Unsupervised machine learning (97)	Clustering (79)	
			Unsupervised learning not specified (18)	
		Reinforcement learning (18)	Reinforcement learning (18)	
		Neural networks, including deep learning (392)	Deep learning (223)	
			Deep neural networks (31)	
			Artificial neural networks (20)	
			Convolutional neural networks (86)	
			Recurrent neural networks (4)	
			Generative adversarial networks (3)	
			Long–short-term memory networks (4)	
			Neural networks not specified (36)	
		Pattern recognition (46)		
		Machine learning not specified (398)		
	Perception	Natural language processing (59)	Natural language processing (42)	
			Sentiment analysis (5)	
			Chatbot (17)	
		Computer vision (78)	Computer vision (43)	
			Machine vision (4)	
			Image recognition (45)	
Integration	Robotics (111)		Robotics (111)	
Other	Artificial intelligence not specified (449)			

* More than one choice possible.

**Table 6:** Disease areas of AI-related trials by ICD-11.

Classification *	Number of Trials (*N* = 1573)	Percent (%) **
Neoplasms	392	24.92
Diseases of the circulatory system	204	12.97
Diseases of the nervous system	181	11.51
Diseases of the digestive system	139	8.84
Mental, behavioural, or neurodevelopmental disorders	136	8.65
Symptoms, signs, or clinical findings not elsewhere classified	112	7.12
Endocrine, nutritional, or metabolic diseases	101	6.42
Diseases of the musculoskeletal system or connective tissue	89	5.66
Diseases of the visual system	78	4.96
Factors influencing health status or contact with health services	74	4.70
Certain infectious or parasitic diseases	70	4.45
Injury, poisoning, or certain other consequences of external causes	69	4.39
Codes for special purposes (RA01, RA02) ***	66	4.20
Diseases of the respiratory system	63	4.01
Diseases of the genitourinary system	48	3.05
Diseases of the immune system	21	1.34
Developmental anomalies	18	1.14
Sleep–wake disorders	17	1.08
Pregnancy, childbirth, or the puerperium	17	1.08
Certain conditions originating in the perinatal period	11	0.70
Diseases of the blood or blood-forming organs	10	0.64
External causes of morbidity or mortality	8	0.51
Diseases of the skin	8	0.51
Diseases of the ear or mastoid process	6	0.38

* More than one choice possible. ** Sum partly ≠ 100 due to multiple choices. *** RA01 code indicates “COVID-19”, RA02 code indicates “post-COVID-19 condition”.

**Table 7:** Healthcare application fields of AI-related trials.

Application *	Number of Trials (*N* = 1725)	Percent (%) **
Diagnosis and screening	662	38.38
Medical imaging	365	21.16
Clinical outcome prediction	296	17.16
Patient monitoring and management	177	10.26
Clinical decision support	155	8.99
Adjuvant treatment	108	6.26
Surgery	95	5.51
Rehabilitation	78	4.52
Biomarker discovery	56	3.25
Analysis of disease risk factors	46	2.67
Patient identification and risk stratification	46	2.67
Health management	32	1.86
Living assistance	28	1.62
Mechanism research	25	1.45
Other	46	2.67

* More than one choice possible. ** Sum partly ≠ 100 due to multiple choices.

## Data Availability

The data supporting this research were from the ClinicalTrials.gov website [31], available online: https://clinicaltrials.gov/ (accessed on 15 July 2022).

## Supplementary Materials

The following supporting information can be downloaded at: https://www.mdpi.com/article/10.3390/ijerph192013691/s1.
