# Supplementary material for: Characteristics of Artificial Intelligence Clinical Trials in the Field of Healthcare: A Cross-Sectional Study on ClinicalTrials.gov
Source: Int J Environ Res Public Health. 2022 Oct 21;19(20):13691. doi: 10.3390/ijerph192013691 (PMC9602501; doi:10.3390/ijerph192013691)
Supplement: Supplementary file 1 [file ijerph-19-13691-s001.zip › ijerph-1933279-supplementary.pdf]

International Journal of
Environmental Research and Public Health

MDPI

Characteristics of artificial intelligence clinical trials in the field of healthcare: A Cross-Sectional Study on ClinicalTrials.gov

Table S1. Characteristics of completed interventional trials by the status of results reporting.

Characteristics	Number (%) of studies (*N* = 249)	
	Reported (*n* = 27)	Not reported (*n* = 222)
**Posted year**		
Before 2007	2 (7.41)	4 (1.80)
2008-2012	6 (22.22)	18 (8.11)
2013-2017	14 (51.85)	57 (25.68)
After 2018	5 (18.52)	143 (64.41)
**Status**		
Completed	22 (81.48)	188 (84.68)
Suspended	0 (0)	3 (1.35)
Terminated	5 (18.52)	10 (4.50)
Withdrawn	0 (0)	21 (9.46)
**Allocation**		
Randomized	7 (25.93)	114 (51.35)
Non-randomized	3 (11.11)	23 (10.36)
NA	17 (62.96)	83 (37.39)
**Masking**		
None/open label	27 (100)	143 (64.41)
Single	0 (0)	46 (20.72)
Double or more	0 (0)	33 (14.86)
**Phase**		
Phase 1	0 (0)	6 (2.70)
Phase 1/Phase 2	1 (3.70)	3 (1.35)
Phase 2	1 (3.70)	5 (2.25)
Phase 2/Phase 3	0 (0)	1 (0.45)
Phase 3	1 (3.70)	3 (1.35)
Phase 4	1 (3.70)	6 (2.70)
NA	23 (85.19)	198 (89.19)
**Enrollment**		
≤100	17 62.96)	134 (60.36)
>100	10 (37.04)	88 (39.64)
**Center**		
Single-center	15 (55.56)	194 (87.39)
Multi-center	12 (44.44)	28 (12.61)
**Lead sponsor**		
Hospital/University	12 (44.44)	132 (59.46)
Industry	8 (29.63)	50 (22.52)
Other	7 (25.93)	40 (18.02)
**Region of lead sponsor**		
North America	22 (81.48)	109 (49.10)
Europe	3 (11.11)	61 (27.48)

Asia	1 (3.70)	47 (21.17)
Other	1 (3.70)	5 (2.25)
**Funded by**		
NIH/US Fed	8 (29.63)	17 (7.66)
Industry/other	19 (70.37)	205 (92.34)

Abbreviations: NA, not applicable; NIH, National Institutes of Health; US Fed, other United States Federal Agencies.

Table S2. Description of entity types in the AI technology application graph.

Entity types	Description	Example
AI_subdomain	classfication of AI subdomains	e.g., supervised machine learning
AI_technology	detailed AI technologies of different subdomains	e.g., support vector machine
condition	classification of conditions by ICD11	e.g., neoplasms
healthcare_setting	classification of healthcare application settings	e.g., diagnosis and screening

Table S3. Description of entity relationship types in the AI technology application graph.

Relationship types	Description	Example
has_a	AI subdomain contains detailed AI algorithms or models	e.g., <“supervised machine learning” - has_a - “support vector machine”>
is_applied_to	AI technology can be apply to the healthcare scenarios	e.g., <“support vector machine” -is_applied_to - “diagnosis and screening”>
focus_on	focused disease areas in healthcare scenarios	e.g., <“diagnosis and screening” -focus_on - “neoplasms”>

Table S4. Statistics on the application of deep learning technology (list the top 10 items).

Rank	AI technology	Healthcare setting	Conditions	Number of trials
1	Deep learning	Diagnosis and screening	Neoplasms	54
2	Deep learning	Diagnosis and screening	Diseases of the digestive system	20
3	Deep learning	Diagnosis and screening	Diseases of the visual system	18
4	Deep learning	Diagnosis and screening	Symptoms, signs or clinical findings, not elsewhere classified	10
5	Deep learning	Diagnosis and screening	Diseases of the circulatory system	9
6	Deep learning	Diagnosis and screening	Other conditions not classified	9
7	Deep learning	Diagnosis and screening	Diseases of the nervous system	7
8	Deep learning	Diagnosis and screening	Endocrine, nutritional or metabolic diseases	4
9	Deep learning	Diagnosis and screening	Diseases of the genitourinary system	4
10	Deep learning	Diagnosis and screening	Mental, behavioural or neurodevelopmental	3

	disorders
